# Copper Homeostasis and Cuproptosis in Neurodegenerative Diseases

**DOI:** 10.3390/cells15141238

**Published:** 2026-07-09

**Authors:** Bowen Liu, Lingyun Zhang, Bing Lv, Chunjie Xu, Luyu Han, Qiupeng Yan, Xin Wang

**Affiliations:** 1 School of Clinical Medicine, Shandong Second Medical University, Weifang 261053, China; 16653673387@163.com; 2 School of Basic Medical Sciences, Shandong Second Medical University, Weifang 261053, China; zly1993@sdsmu.edu.cn (L.Z.); 15725389360@163.com (B.L.); xu1261781793@163.com (C.X.); 18535582002@163.com (L.H.); 3 Department of Neurosurgery, Brigham and Women’s Hospital, Harvard Medical School, Boston, MA 02115, USA

**Keywords:** cuproptosis, copper homeostasis, amyotrophic lateral sclerosis, Alzheimer’s disease, Parkinson’s disease

## Abstract

Copper is an essential trace element required for numerous enzymatic processes in the brain, including mitochondrial metabolism, antioxidant defense, and gene expression regulation. Recent studies have further implicated copper in a newly defined form of regulated cell death termed cuproptosis, providing a mechanistic framework for copper-dependent cytotoxicity. Increasing evidence indicates that copper dyshomeostasis is a common feature of major neurodegenerative diseases, including Alzheimer’s disease (AD), Parkinson’s disease (PD), and amyotrophic lateral sclerosis (ALS), where it is associated with protein misfolding, redox imbalance, and neuronal vulnerability. Nevertheless, the mechanistic link between copper dysregulation and neuronal cell death remains incompletely defined. In this review, we systematically summarize the molecular mechanisms governing copper homeostasis and intracellular copper trafficking, while providing a timely, updated, and in-depth overview of the mechanistic basis and emerging biology of cuproptosis. We further comprehensively evaluate the current evidence linking copper dysregulation and cuproptosis-related pathways to neurodegenerative diseases, with particular emphasis on distinguishing mechanistic causation from pathological correlation. Importantly, we discuss current therapeutic strategies and emerging clinical efforts targeting copper metabolism, while highlighting the major challenges in defining the pathological significance and mechanistic contribution of cuproptosis in neurodegenerative diseases. Collectively, this review provides an updated framework for understanding the pathological significance and translational potential of cuproptosis in neurodegenerative diseases.

## 1. Introduction

Neurodegenerative diseases, including Alzheimer’s disease (AD), Parkinson’s disease (PD), and amyotrophic lateral sclerosis (ALS), are characterized by progressive neuronal loss and impose a substantial and growing global health burden [1,2,3]. Disrupted metal ion homeostasis has emerged as a common pathological feature of these disorders [4]. Among essential trace metals, copper occupies a unique position due to its dual role as an indispensable catalytic cofactor and a potential mediator of cellular toxicity [5].

Copper is required for multiple fundamental biological processes, including mitochondrial respiration, antioxidant defense, neurotransmitter synthesis, and extracellular matrix remodeling. To sustain these functions while preventing cytotoxicity, intracellular copper levels are tightly controlled by coordinated networks of transporters, chaperones, and storage systems [6]. Dysregulation of copper homeostasis has been consistently observed across neurodegenerative diseases and is mechanistically linked to protein misfolding, redox imbalance, and increased neuronal vulnerability [7,8]. However, how copper dysregulation is coupled to neuronal cell death remains incompletely defined.

Recently, cuproptosis has been identified as a novel form of regulated cell death, providing a mechanistic framework for copper-dependent cytotoxicity. Distinct from canonical oxidative stress-driven cell death pathways, cuproptosis is mediated by the direct binding of copper to lipoylated enzymes of the tricarboxylic acid (TCA) cycle, thereby inducing protein aggregation and destabilization of iron–sulfur (Fe-S) cluster proteins [7,9,10]. Notably, this process is tightly coupled to mitochondrial metabolic activity, rendering cells with high oxidative phosphorylation dependency particularly susceptible [9].

Given the central role of mitochondrial metabolism in neuronal function, cuproptosis has attracted increasing attention in the context of neurodegeneration [11,12]. However, current evidence remains largely indirect, and most studies reflect alterations in copper homeostasis or dysregulation of cuproptosis-related genes rather than direct evidence of cuproptotic cell death. Therefore, distinguishing between perturbation of the cuproptosis machinery and bona fide activation of cuproptosis is critical for accurate mechanistic interpretation [9,13].

In this review, we systematically summarize the molecular basis of copper homeostasis and intracellular copper trafficking and provide an updated overview of the mechanistic features of cuproptosis. We further evaluate current evidence linking copper dysregulation to AD, PD, and ALS, with a particular focus on delineating correlation versus causation. Finally, we discuss the therapeutic potential of targeting copper metabolism and highlight key challenges that must be addressed to clarify the pathological relevance of cuproptosis in neurodegenerative diseases.

## 2. Copper Homeostasis

### 2.1. Absorption, Transport, and Efflux of Copper Ions

**Figure 1:**
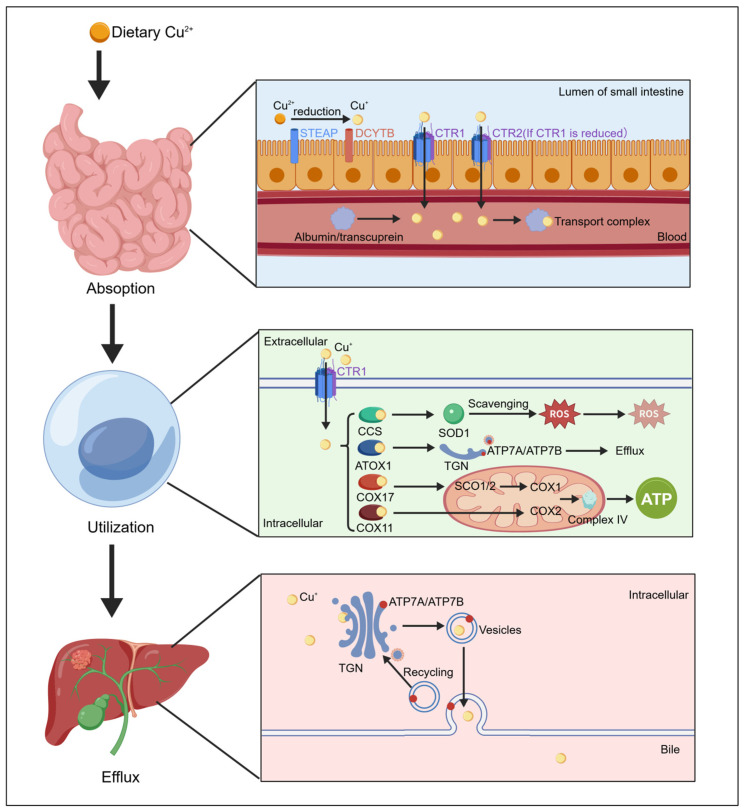
Copper ion transport, absorption, and utilization. Dietary copper is absorbed through the small intestine, mainly the duodenum. Dietary copper is mainly Cu^2+^, which is reduced to Cu^+^ by STEAP and DCYTB on the surface of small intestinal epithelial cells, and then absorbed into the blood by CTR1. CTR2 could transport Cu^+^ in a compensatory manner if the quantity of CTR1 decreased. After entering the blood, it combines with albumin and transcuprein to form a transport complex and is transported to various organs. Cu^+^ can act in three ways in cells. First, Cu^+^ binds to CCS and assists in transporting Cu^+^ to SOD1 to remove ROS from the body. Secondly, it is transported to the TGN under the action of ATOX1, where ATP7A and ATP7B participate in the copper efflux process. Third, COX17 first binds to SCO1 and SCO2 of the mitochondrial inner membrane, then inserts Cu^+^ into the CuB site of COX1 with the assistance of COX16 and COA6, and COX11 directly inserts Cu^+^ into the CuA site of COX2. COX1 and COX2 together form complex IV. It is involved in the process of ATP production. Copper efflux mainly occurs in the liver. When the intracellular Cu^+^ content is too high, ATP7A and ATP7B on the TGN membrane will be translocated to the vesicles, mediating Cu^+^ into the vesicles and fusion with the cell membrane, releasing Cu^+^ into the bile and out of the body with the feces. This figure was created by the authors using BioGDP.com based on the literature cited in this review. In this figure, arrows indicate the direction of regulation or transport. Different colors are used to distinguish distinct cellular compartments, molecular pathways, and specific proteins as indicated in the figure.

Copper undergoes tightly regulated absorption, intracellular trafficking, mitochondrial utilization, and cellular export, ensuring adequate supply while preventing toxic accumulation (Figure 1). Dietary intake is the primary source of copper ions in the human body. Copper in food is mostly in the divalent state (Cu^2+^) and cannot be directly taken up and utilized by intestinal cells. It requires the action of the six-transmembrane epithelial antigen of the prostate and duodenal cytochrome B to reduce Cu^2+^ to monovalent copper ions (Cu^+^) for absorption [14,15]. The primary site of copper ion absorption is the small intestine, particularly the duodenum. Subsequently, copper ions are released into the bloodstream and, with the assistance of soluble molecular chaperones in the blood (such as albumin, transcuprein, macroglobulin, ceruloplasmin [16,17,18]), are transported first to the liver and kidneys [5]. Copper ions from the liver are secreted into the bile via ATPase copper-transporting β polypeptide (ATP7B) and eventually eliminated from the body (Figure 1, left panel).

Dietary copper and unbound copper ions from the blood can be transported from outside the cell to inside the cell mediated by CTR1 (copper transporter 1, also known as SLC31A1) on the intestinal membrane, and this protein is widely present on the cell surface [19]. CTR1 plays a major role in copper transport; however, CTR2 (copper transporter 2, also known as SLC31A2, Solute Carrier Family 31 Member A2) showed compensatory transport function in the absence of CTR1 [19,20]. Therefore, the CTR family is crucial for cellular copper uptake, especially CTR1. After Cu^+^ is taken up by cells, it is transported and distributed to different cellular compartments by various intracellular proteins to exert its normal physiological functions (Figure 1, top panel).

The excretion of cellular copper ions is mediated by ATPase copper-transporting α polypeptide (ATP7A) and ATP7B [21] (Figure 1, bottom panel). ATP7A is distributed in most tissues, while ATP7B is specifically expressed in the liver [22]. ATP7A and ATP7B are located in the trans-Golgi Network (TGN) under normal physiological conditions and mediate the transport of Cu^+^ from the cytoplasm to the TGN lumen, where it is loaded onto cuproenzymes. Under conditions of elevated copper ion concentration, they traffic from the TGN to vesicles and mediate the copper efflux process [23]. Antioxidant 1 copper chaperone (ATOX1) is required for the intracellular transport of copper ions excreted by ATP7A [24]. When copper is excreted, the vesicle-localized Cu-ATPases are recycled back to the TGN and continue to mediate their physiological functions [23]. Current research indicates that abnormalities in ATP7A and ATP7B lead to Menkes disease and Wilson disease, respectively [25,26]. Menkes disease is a rare infantile genetic disorder caused by an ATP7A mutation, leading to reduced ability to absorb copper ions and resulting in copper deficiency in the body [26]. Wilson’s disease is a multi-organ disorder affecting brain tissue due to an ATP7B abnormality, causing excessive copper accumulation [25].

Under the action of ATP7B in hepatocytes, excess copper in the body is further secreted into bile and excreted in feces [5], only a small fraction of which is reabsorbed by the gastrointestinal tract via the CTR1 pathway [27]. This is the primary pathway for copper metabolism in the body. Other excretion pathways, such as urine and sweat, play relatively minor roles in bodily copper metabolism [28].

Although excess free copper ions in cells or the bloodstream can lead to ROS production and cytotoxicity, causing cell death, in tissue cells, these excess copper ions are sequestered through chelation by metallothioneins, a family of proteins capable of binding various heavy metal ions in the body, and glutathione [29]. Studies show that, besides binding copper transported by CTR1, metallothioneins can also indirectly inhibit the ATP7A-mediated copper transport process [30]. Studies have demonstrated that combined deficiency of ATP7A and metallothioneins reduces tolerance to copper-induced cytotoxicity [31,32,33]. Severe Cu overload can also be induced at very low Cu ion concentrations, leading to ROS accumulation, mitochondrial membrane potential collapse, and glutathione (GSH) redox system disruption [30]. Similar copper-mediated oxidative cytotoxicity and cell death mechanisms have also been reported in *Escherichia coli* exposed to copper-phenanthroline complexes [34]. GSH is an important antioxidant in the body. This tripeptide plays an initial buffering role in eliminating aberrant copper ions [35] and it can also be used as a storage form of copper [36].

### 2.2. Intracellular Trafficking and Functions of Copper

Intracellular copper levels are tightly regulated by a network of copper chaperones and transporters that ensure precise delivery to target proteins, thereby orchestrating multiple key cellular processes, including antioxidant defense, neurotransmitter synthesis, and mitochondrial respiration (Figure 1, middle panel).

Copper chaperone for SOD1 (CCS) can bind Cu^+^ and transport it to copper-zinc superoxide dismutase 1 (SOD1), where it functions to scavenge reactive oxygen species (ROS) and maintain copper homeostasis. SOD1 functions in the cytoplasm, eliminating cellular oxidative stress by converting intracellular superoxide radicals into oxygen or hydrogen peroxide [34,37]. Similarly, CCS and SOD1 are localized in the cytoplasm and the mitochondrial intermembrane space. Therefore, intracellular SOD1 plays an important role in scavenging free radicals and protecting cells. Furthermore, CCS is a cellular transcription factor regulated by copper concentration. When intracellular copper content is high, CCS degradation increases; when intracellular copper content is low, CCS synthesis increases [38,39].

Cu^+^ can also bind to ATOX1 and be transported to the ATP7A and ATP7B on the trans-face of the Golgi network [40], further promoting the synthesis of cuproenzymes (such as lysyl oxidase, tyrosinase, ceruloplasmin, etc.) [22,26]. Moreover, ATOX1 belongs to a class of copper-dependent transcription regulators. Studies show that ATOX1 favors cell proliferation. ATOX1 knockout mice experience disrupted copper homeostasis, leading to perinatal death, reflecting the central role of ATOX1 in copper transport and homeostasis [9].

Mitochondria have been implicated as key organelles involved in intracellular copper utilization and storage (Figure 1, middle). Mitochondrial copper is primarily required for the metallation of cytochrome c oxidase (COX; Complex IV) and SOD1, both of which are essential for oxidative metabolism and redox homeostasis. Cytosolic copper is thought to enter mitochondria as a low-molecular-weight anionic ligand complex (CuL). Copper-bound CuL is transported into the mitochondrial intermembrane space, possibly through solute carrier family 25 member 3 (SLC25A3), where it serves as a dynamic copper reservoir capable of adjusting to cellular copper availability and ensuring a continuous copper supply [41].

After entering mitochondria, copper, as a cofactor of COX, is directly involved in the generation of proton motive force and the complete reduction of oxygen to water, and can regulate the respiration rate by modulating the assembly and disassembly of COX [41]. COX itself acts as a metal sensor: copper deficiency reduces COX expression and activity, inhibits respiration and ATP production, and causes electron transport chain remodeling, whereas sufficient copper promotes COX assembly and drives the increase in other complexes such as complex I, which overall enhances oxidative phosphorylation [41].

At the same time, copper is a cofactor of SOD1, endowing it with the activity to scavenge superoxide anions and thereby alleviate reactive oxygen species produced by the electron transport chain [42]. In addition, copper also acts as a cofactor of ferroxidase, indirectly affecting mitochondrial iron uptake; since iron transport is critical for iron-sulfur cluster assembly and heme biosynthesis, copper can thereby indirectly regulate mitochondrial function through the iron metabolic pathway [43].

Within mitochondria, copper trafficking to COX requires a highly coordinated chaperone system. The transport of copper into mitochondria primarily relies on cytochrome c oxidase copper chaperone 17 (COX17), which transfers Cu^+^ from the cytoplasm to two proteins on the mitochondrial inner membrane: Synthesis of cytochrome c oxidase 1 (SCO1) and Synthesis of cytochrome c oxidase 2 (SCO2), thereby promoting copper insertion into the mitochondrially encoded cytochrome c oxidase subunit 2 (COX2, also known as MT-CO2) [44]. The transfer of copper from SCO1 and SCO2 to COX2 is catalyzed and facilitated by two enzymes: cytochrome c oxidase assembly protein 16 (COX16) and cytochrome c oxidase assembly factor 6 (COA6) [45,46]. Another pathway involves copper transport from the cytoplasm to cytochrome c oxidase subunit 1 (COX1, also known as MT-CO1) mediated by cytochrome c oxidase assembly protein 11 (COX11) [47]. The redox-active copper centers in COX1 and COX2 play a key role in the electron transfer process of Complex IV, with Cu^+^ binding to the CuA site of COX2 and the CuB site of COX1, respectively [48].

Copper homeostasis in the nervous system exhibits several features that distinguish it from other organs. During neuronal differentiation, intracellular copper levels rise significantly, with increased copper flow specifically directed to the secretory pathway rather than the cytosolic compartment, a process regulated by the GSH/GSSG redox balance via ATOX1 [49,50,51]. The copper transporter ATP7A is expressed in a developmental rather than constitutive manner, peaking prior to synaptogenesis, and its axonal localization is critical for neurite extension and synapse formation [52,53]. Within neurons, ATP7A and ATP7B exhibit distinct subcellular distributions: ATP7B is largely restricted to the somatodendritic compartment, whereas ATP7A clusters at the axonal side of the nucleus, and copper elevation or NMDA receptor activation drives their redistribution to axons [54,55,56]. Neurons also utilize copper for neurotransmitter and neuropeptide synthesis through the cuproenzymes dopamine-β-hydroxylase (DBH) and peptidylglycine α-amidating monooxygenase (PAM), which are essential for norepinephrine production and neuropeptide maturation, respectively [57,58]. Moreover, copper is released from presynaptic terminals upon depolarization, or NMDA receptor activation, and this synaptically released copper modulates ionotropic receptors—including NMDA, AMPA, and GABAA receptors—thereby influencing network excitability and providing neuroprotection against excitotoxicity [59,60]. Beyond synaptic transmission, copper regulates spontaneous network activity in the hippocampus and retina, phase-shifts circadian rhythms in the suprachiasmatic nucleus via NMDAR-dependent mechanisms and affects rest-activity cycles and arousal through the locus coeruleus–norepinephrine system [58,61]. Finally, specific neuronal subpopulations, particularly motor neurons, display heightened vulnerability to ATP7A dysfunction, as evidenced by distal hereditary motor neuropathy and ALS-linked ATP7A variants, while in Wilson disease, copper accumulation preferentially damages the basal ganglia, thalamus, cerebellum, and brainstem [62,63]. These neuron-specific features underscore the particular susceptibility of the nervous system to copper dyshomeostasis.

## 3. Cuproptosis

### 3.1. Characteristics of Cuproptosis

Cuproptosis has been reported as a novel form of regulated cell death, characterized by the loss of Fe-S clusters and the aggregation of fatty acylated proteins, which leads to dysfunction of the TCA cycle in various cell types and further causes intracellular oxidative damage [64,65]. Cuproptosis is strictly dependent on copper ions; research found that using a low concentration (40 nM) of the copper ionophore elesclomol can induce cuproptosis in human cell lines [66].

Compared to other forms of regulated cell death, cuproptosis exhibits unique morphological and biochemical hallmarks. The most prominent morphological change is mitochondrial alteration, manifested as mitochondrial swelling and cristae fragmentation [42,67]. The latest research shows that the mitochondrial performance of cells undergoing cuproptosis is often similar to that of ferroptosis. Cristae deformation, mitochondrial shrinkage, separation of inner and outer membranes, increased density of matrix or replacement by vacuoles, and vesicles formed by mitochondria surrounding rough endoplasmic reticulum were observed during cuproptosis [68]. The resistance of cuproptosis to conventional cell death inhibitors further underscores its uniqueness. Treatment with ferrostatin-1 (ferroptosis inhibitor), necrostatin-1 (necroptosis inhibitor), N-acetylcysteine (inhibits oxidative stress), and Z-VAD-FMK (apoptosis inhibitor) all failed to reverse the cuproptosis process induced by elevated intracellular copper concentration [66]. Conversely, only copper chelators can rescue cuproptosis triggered by increased intracellular copper levels [66]. These characteristics indicate that cuproptosis is a distinct form of regulated cell death.

A hallmark feature of cuproptosis is its impact on mitochondrial metabolism, particularly affecting TCA cycle enzymes. Research revealed progressive TCA dysfunction upon treatment with copper ionophores [66]. Studies have shown that cells that rely on mitochondria for respiration are approximately 1000-fold more sensitive to cuproptosis than those that utilize glycolysis [66]. The use of complex I inhibitors significantly inhibited the process of cuproptosis in cells, and further studies indicated that the process of cuproptosis was more dependent on mitochondrial respiratory processes rather than ATP production [66].

### 3.2. Molecular Mechanisms of Cuproptosis

Cuproptosis is primarily mediated by Cu^+^. Current evidence suggests that mitochondrial Cu^+^ is supplied through two major routes. One route involves copper ionophores, which shuttle excess extracellular Cu^2+^ into mitochondria, where it is reduced to Cu^+^ by ferredoxin 1 (FDX1). The other route is the direct transport of cytosolic Cu^+^ into mitochondria by SLC25A3. The resulting increase in mitochondrial Cu^+^ provides the copper species that binds lipoylated proteins and initiates cuproptosis.

**Figure 2:**
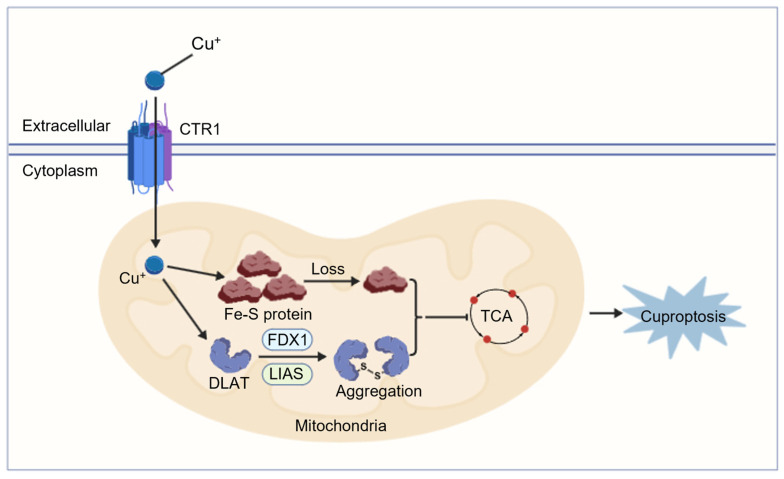
Biological mechanisms of copper poisoning. Extracellular Cu^2+^ is first reduced to Cu^+^ by reductases such as STEAP or DCYTB at the plasma membrane, and Cu^+^ is then imported into the cell by CTR1. Cu^+^ within mitochondria binds to Fe-S cluster proteins and, with the participation of FDX1 and LIAS, causes cluster loss and abnormal DLAT aggregation through disulfide bonds. This eventually disrupts the mitochondrial TCA cycle and induces cuprites. This data was created by the authors on BioGDP.com based on the literature cited in this paper. In this figure, arrows indicate the direction of regulation or transport. Different colors are used to distinguish distinct cellular compartments, molecular pathways, and specific proteins as indicated in the figure.

Excess Cu^+^ induces cuproptosis mainly through two key pathways. When intracellular Cu^+^ concentration is abnormally elevated, excess copper ions bind to lipoylated proteins within the mitochondria, leading to their abnormal aggregation (Figure 2) [66]. Lipoylation is a highly conserved type of modification. Although currently relatively rare, existing research indicates that all lipoylated proteins participate in the cellular TCA cycle process [69,70]. Further research revealed that Cu^+^ treatment significantly increases the degree of mitochondrial lipoylation in human cell lines, particularly in four enzymes: DBT (dihydrolipoamide branched chain transacylase), GCSH (glycine cleavage system H protein), DLST (dihydrolipoamide S-succinyltransferase), and DLAT (dihydrolipoamide S-acetyltransferase) [7]. Among these, DLAT is a subunit of the pyruvate dehydrogenase complex [71] and has been identified as one of the targets of copper in lipoyltransferases. Unbound copper mediates the disulfide bond-dependent aggregation of DLAT, thereby affecting the TCA cycle process [71]. In addition, the buildup of aggregated lipoylated proteins creates proteotoxic stress, which worsens mitochondrial injury [72].

The other pathway involves free Cu^+^ promoting cuproptosis by disrupting Fe-S cluster proteins (Figure 2). Fe-S clusters are essential cofactors for numerous mitochondrial proteins, including components of the electron transport chain such as NADH dehydrogenase and succinate dehydrogenase. Loss of Fe-S clusters impairs electron transport, reduces ATP production, and disrupts the mitochondrial membrane potential [73]. Consequently, mitochondrial dysfunction leads to increased inner mitochondrial membrane permeability, calcium overload within the mitochondrial matrix, and excessive production of ROS [72]. Studies in yeast have shown that excess copper inhibits cell growth by damaging the labile Fe–S cluster protein pool, thereby disrupting Fe–S cluster homeostasis [74]. In vitro, copper also inhibits Fe-S cluster protein formation by inhibiting mitochondrial assembly protein activity [75].

The combined effects of lipoylated protein aggregation and Fe–S cluster disruption culminate in mitochondrial dysfunction and proteotoxic stress, ultimately triggering cuproptotic cell death [76].

### 3.3. The Regulation of Cuproptosis

Current evidence identifies FDX1 as a central regulator of cuproptosis. In addition to reducing Cu^2+^ to the cuproptosis-inducing Cu^+^ within mitochondria, FDX1 plays a critical role in regulating protein lipoylation [7]. FDX1 promotes the lipoylation of key TCA cycle enzymes, thereby generating the lipoylated protein substrates required for copper binding and aggregation during cuproptosis [66]. Consistently, FDX1 knockdown reduces intracellular pyruvate and α-ketoglutarate metabolism, decreases the abundance of lipoylated proteins, and markedly suppresses cuproptosis in both cancer and human cell lines [10,66]. In addition, recent studies suggest that FDX1 is also involved in the copper-induced destabilization of iron–sulfur (Fe–S) cluster proteins, although the underlying mechanism remains incompletely understood [77].

Lipoic acid synthetase (LIAS) is another key regulator of cuproptosis and serves as an essential enzyme in the mitochondrial protein lipoylation pathway [10,66]. LIAS catalyzes the lipoylation of mitochondrial proteins, generating the lipoylated substrates required for copper binding and the initiation of cuproptosis [66,69]. Similar to FDX1 deficiency, LIAS knockdown markedly reduces protein lipoylation and inhibits cuproptosis, further highlighting the essential role of the mitochondrial lipoylation pathway in this form of regulated cell death [10,66]. LIAS catalyzes the lipoylation of mitochondrial proteins, generating the lipoylated substrates required for copper binding and the initiation of cuproptosis [77].

The sensitivity of cuproptosis is subject to multiple molecular regulations but is independent of oxidative stress. Glutathione blocks cuproptosis by chelating intracellular copper, whereas other antioxidants such as N-acetylcysteine and α-tocopherol fail to do so, indicating that reactive oxygen species are not essential executioners of cuproptosis. At the metabolic level, mitochondrial aerobic respiration is a core driver of cuproptosis: cells relying on oxidative phosphorylation are highly sensitive, and inhibition of respiratory chain complexes I and III, blockade of mitochondrial pyruvate uptake, or hypoxia can effectively restrict cuproptosis, distinguishing it from ferroptosis, which requires glucose uptake and pyruvate oxidation in cell culture models [78,79]. In terms of copper homeostasis, overexpression of the copper importer SLC31A1 enhances cuproptosis, while deletion of the copper exporter ATP7B results in copper accumulation and cuproptotic damage; inhibition of GSH synthesis also increases susceptibility to cuproptosis [66].

Mitochondrial quality control mechanisms such as mitophagy, along with protein degradation systems including autophagy and the ubiquitin–proteasome system, may limit cuproptosis, although their precise regulatory roles remain to be clarified [80].

Aging is intrinsically linked to the disruption of copper homeostasis, which arises from reduced biliary excretion, impaired brain copper clearance at the blood-cerebrospinal fluid barrier, and diminished cellular copper-buffering capacity due to declines in glutathione and metallothionein levels [81,82]. This age-related copper overload creates a permissive environment for cuproptosis, a recently identified form of regulated cell death initiated by the direct binding of excess copper to lipoylated mitochondrial proteins-particularly dihydrolipoamide S-acetyltransferase (DLAT)-leading to their aggregation, loss of iron-sulfur cluster proteins, and proteotoxic stress, a process critically dependent on ferredoxin 1 (FDX1) [66]. Importantly, cuproptosis is not merely a consequence of aging but actively accelerates the aging process by exacerbating several canonical hallmarks. It promotes mitochondrial dysfunction through respiratory chain impairment and reduced ATP production [83], while simultaneously increasing oxidative stress via Fenton-type generation of reactive oxygen species [84]. Furthermore, copper overload disrupts proteostasis by inhibiting the ubiquitin-proteasome system and impairing autophagic clearance, leading to the accumulation of misfolded proteins [85,86]. It also fuels chronic inflammation by triggering the release of damage-associated molecular patterns such as HMGB1 and activating the cGAS-STING pathway, thereby sustaining a pro-inflammatory milieu [87,88]. Additionally, copper dyshomeostasis has been linked to telomere shortening and epigenetic alterations, including inhibition of S-adenosylhomocysteine hydrolase, which further drives cellular senescence [89]. This bidirectional relationship—where aging promotes copper accumulation and cuproptosis, which in turn amplifies age-related degenerative processes—establishes a vicious cycle that underlies the pathogenesis of various age-related diseases, including neurodegenerative disorders, cardiovascular diseases, and metabolic syndromes. Targeting copper homeostasis and cuproptosis pathways, through chelators, natural products, or gene therapy, therefore holds therapeutic promise for mitigating age-associated pathologies [90,91].

## 4. Copper and Alzheimer’s Disease

### 4.1. Copper Homeostasis and Cuproptosis in Alzheimer’s Disease

AD is a prevalent neurodegenerative disease primarily affecting the elderly. AD involves abnormal processing of the amyloid precursor protein in brain gray matter cells, leading to the deposition of amyloid plaques and neurofibrillary tangles [3]. At the molecular level, AD is characterized by the aggregation of the amyloid-β (Aβ) peptide and tau protein [92]. Growing evidence indicates that abnormal copper homeostasis is closely related to the pathogenesis of AD (Table 1). Studies show that both free and total copper levels are increased in the serum of AD patients, with the free copper level in serum and brain increasing with age [93,94,95]. High concentrations of copper ions have also been detected in the senile plaques of AD patients [96]. Furthermore, excessive intake of copper ions or elevated copper ion concentration in the body is associated with memory impairment in patients in a human epidemiological study [97]. Consistent findings have been observed in animal models. In the J20 AD mouse model, copper exposure led to spatial memory decline in the J20 transgenic mouse model of AD [98]. Similarly, in an AD rabbit model, chronic consumption of drinking water containing a low concentration of copper (0.12 ppm) resulted in Aβ accumulation and impaired learning ability in the rabbit model of AD [99]. Furthermore, reducing systemic copper levels in female PS19 mice alleviated spatial memory deficits [100]. The above results indicate that excessive copper exposure can lead to AD symptoms in humans and mice.

Interestingly, copper deficiency may also have detrimental effects. One study demonstrated that low copper concentrations reduced the expression of low-density lipoprotein receptor-related protein 1 (LRP1), thereby impairing Aβ clearance from the BV2 mouse microglial cell line [101,102]. Therefore, both copper overload and copper deficiency may contribute to AD pathogenesis, highlighting the critical role of copper dyshomeostasis in disease progression.

**Figure 3:**
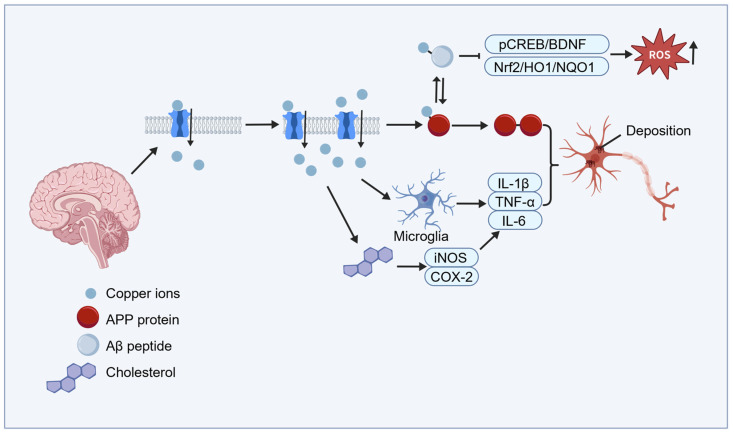
The potential mechanisms by which copper ions affect the progression of AD. Increased CTR1 expression in brain cells leads to increased intracellular copper ion content. Elevated copper may affect the progression of AD through three potential pathways. Firstly, copper ions can bind to APPs, and some of the APPs can directly cross-link into dimers, leading to deposition in neuronal cytoplasm. The copper-binding APP was converted to the Aβ peptide, which promoted ROS production by inhibiting the pCREB/BDNF pathway and the Nrf2/HO1/NQO1 pathway. Second, copper can stimulate microglia to release inflammatory mediators such as IF-1β, TNF-α, and IL-6, thereby promoting neuronal deposition in the cytoplasm. Thirdly, Copper could interact with cholesterol to promote iNOS and COX-2 expression, which is similar to pathway 2. This figure was created by the authors using BioGDP.com based on the literature cited in this review. In this figure, arrows indicate the direction of regulation or transport. Different colors are used to distinguish distinct cellular compartments, molecular pathways, and specific proteins as indicated in the figure.

Copper dysregulation contributes to AD through several mechanisms, including Aβ pathology, tau pathology, oxidative stress, and neuroinflammation (Figure 3). Among these, the interaction between copper ions and Aβ peptides has been extensively investigated and is considered a key event in AD pathogenesis. Aβ peptides are generated through sequential cleavage of amyloid precursor protein (APP) by β-secretase and γ-secretase [96]. APP has Cu^2+^ binding sites; binding to copper ions promotes the dimerization of APP, leading to increased Aβ peptide deposition in patient brains [96]. In addition, studies show that under copper exposure conditions, the expression of the gene for β-site APP cleaving enzyme 1 (BACE1) is promoted in 3×Tg-AD transgenic mouse model cells, leading to increased intracellular Aβ peptide accumulation [103].

The Aβ peptide itself also has Cu^2+^ binding sites. The coordination of Cu^2+^ with the histidine residues His6, His13, and His14 at the N-terminal of Aβ drives the conformational transition to β-rich oligomers to form aggregates resistant to protease degradation, thereby accelerating deposition [104]. Research also showed that the formation of Cu^2+^-Aβ peptide complexes promotes senile plaque deposition and cellular ROS production in patients, which further enhances the neurotoxicity of Aβ peptide, ultimately leading to increased oxidative damage [105,106]. In addition, the Cu^2+^-Aβ peptide complex can interact with APP and promote the formation of dityrosine-crosslinked neurotoxic Aβ dimers in vitro biochemical studies [107,108]. Copper binding stabilizes these dimers, making them resistant to dissociation and facilitating amyloid deposition in brain tissue [109]. Furthermore, the Cu^2+^-Aβ peptide complex leads to increased aggregation of unbound Aβ peptide, further exacerbating Aβ accumulation and neurotoxicity in vitro biochemical and cell culture studies [108,110].

Aβ has a higher affinity for Cu^+^ than Cu^2+^. However, the role of Cu^+^ in the aggregation process remains controversial, with some reports suggesting that it can stabilize monomers and inhibit fibrosis after occupying key histidine sites, while other evidence suggests that Cu^+^ may exacerbate pathology by REDOX cycling or inducing toxic oligomerization [111].

**Table 1:** Copper level changes in AD, PD, and ALS.

Disease	Species	Source	Copper Level Change	Reference
AD	Human	Serum	Increased ↑	[93,94,95]
	Human	Brain	Increased ↑	[93,94,95]
	Human	Senile plaques	Increased ↑	[96]
PD	Human	Substantia nigra	Decreased ↓	[112]
ALS	Mouse	Spinal cord	Decreased ↓	[113,114]
	Mouse	Skeletal muscle	Decreased ↓	[113,114]
	Human	cerebrospinal fluid	Increased ↑	[115]
	Human	cerebrospinal fluid	Decreased ↓	[116]

Evidence also indicates that copper interacts with tau, another key pathogenic protein involved in AD [3]. A single tau molecule can bind one Cu^2+^ ion with micromolar affinity, and this interaction promotes the conformational transition of tau into β-sheet-rich fibrillar structures in vitro biochemical studies [117]. Additionally, copper exposure has been shown to accelerate tau pathology in 3×Tg-AD mice, resulting in increased tau phosphorylation [103]. Current research suggests this process is related to the activation of the CDK5/P25 pathway [103]. Furthermore, copper imbalance can activate GSK-3β and inhibit PP2A through oxidative stress, thereby indirectly enhancing tau hyperphosphorylation and subsequent aggregation [118].

Disruption of copper homeostasis (overload or deficiency) can also promote neuroinflammation in AD through distinct pathways (Figure 3). Previous research indicates that copper ions play a regulatory role in maintaining microglial homeostasis [119]. Copper increases the secretion of pro-inflammatory cytokines (such as interleukin-1β (IL-1β), interleukin-6 (IL-6), and tumor necrosis factor-α (TNF-α)) by microglia and impairs phagocytic function. This ultimately leads to reduced Aβ peptide clearance in the BV2 mouse microglial cell line [101]. Further research shows that copper ions cause disruption of the microglial stable phenotype and exacerbate neuroinflammation [98]. Trace copper ions can also bind to cholesterol, leading to the production of high levels of inducible nitric oxide synthase (iNOS) and COX-2 in cells, which further promotes the release of TNF-α and leads to an enhanced inflammatory response in a mouse model [120]. At sub-neurotoxic concentrations, copper ions activate microglia via the NF-κB pathway, leading to increased production of TNF-α and NO, thereby causing neurotoxicity [121], and the Cu^2+^-Aβ complex can promote this pathological process and generate neurotoxicity in microglial cell culture [122]. Copper overload can indirectly drive neuroinflammation through the cuproptosis pathway: microglia increase intracellular copper accumulation by upregulating the copper transporter CTR1 and undergo cuproptosis, releasing pro-inflammatory factors such as IL-1β and TNF-α, mtDNA, activating the NLRP3 inflammasome in astrocytes, and promoting chronic neuroinflammation [82] (Figure 3).

Copper ions also impact cellular oxidative stress (Figure 3). Studies show that free copper ions can induce the production of ROS in the human brain [94,123]. Simultaneously, copper ions participate in cellular ROS production and lipid peroxidation processes, leading to reduced activity of antioxidant enzymes in hippocampal neuron cell culture [124]. The action of the Cu^2+^-Aβ complex leads to increased hydrogen peroxide release and enhanced mitochondrial ROS generation [122]. Moreover, this complex can inhibit the pCREB/brain-derived neurotrophic factor (BDNF) and nuclear factor erythroid 2-related factor 2 (Nrf2)/heme oxygenase-1 (HO-1)/NAD(P)H quinone dehydrogenase 1 (NQO1) pathways and exacerbate oxidative stress in cells [122]. Copper dyshomeostasis, on the other hand, indirectly exacerbates oxidative stress by impairing the function of several copper-dependent enzymes. Reduced activities of SOD1 and cytochrome c oxidase weaken antioxidant defenses and compromise mitochondrial function in the AD rat model [125].

Several cuproptosis-related genes are also involved in AD pathogenesis (Table 2). CTR1 (also known as SLC31A1) is an important protein responsible for copper ion transport in cells. This protein plays a significant role in regulating the copper ion concentration in tissue cells and in cuproptosis. Studies have shown that knockdown of CTR1 significantly reduces the concentration of copper in the brain of patients, alleviates the neurodegenerative changes induced by Aβ peptide, and enhances the resistance of cells to oxidative damage [126]. LIAS is involved in cellular mitochondrial energy metabolism and antioxidant processes, and its deficiency can lead to mitochondrial energy metabolism defects [69,127]. LIAS is involved in the biosynthesis of lipoic acid (LA) in cells, which is ultimately processed to alpha-lipoic acid (ALA) within mitochondria. ALA is an important molecule in the process of energy metabolism and antioxidation. ALA can reduce the generation of ROS and improve mitochondrial function [69,70]. ALA has been shown to protect mitochondria from oxidative stress and ameliorate mitochondrial dysfunction in AD cell models [128]. ALA supplementation enhances cognitive function in AD patients and aged Tg2576 mice [129,130]. In addition, ALA reduces iron overload, lipid peroxidation, and inflammation associated with AD and can improve cognitive impairment in P301S Tau transgenic mice [131]. Overall, LIAS further influences AD progression by affecting ALA concentrations.

Although the causal relationship between cuproptosis and AD remains unclear, abnormal expression of cuproptosis-related genes has been observed in AD. A recent study analyzed the expression profiles of cuproptosis-related genes (CRGs) in the brains of AD patients and healthy individuals. The research team first obtained 13 CRGs from Tsvetkov’s research [66]. Among the 13 genes, CTR1, ATP7B, LIPT1, CDKN2A, and MTF1 were upregulated in the cerebral cortex of AD patients [132], where the expression of FDX1, DLAT, GLS, PDHA1, DLD, PDHB, and LIAS was downregulated [132], indicating that cuproptosis is potentially dysregulated in the context of AD.

### 4.2. Copper-Related Therapeutic Strategies in AD

In AD-related research, some copper chelators have shown good effects in treating AD (Table 3). Clioquinol, a drug that can cross the blood–brain barrier, was shown to inhibit copper ion-induced Aβ peptide aggregation. Results from a small-scale clinical study demonstrated that AD patients exhibited slight improvements in clinical assessment scores after three weeks of clioquinol treatment, suggesting a potential therapeutic effect [134,135]. Simultaneously, it can also reduce BACE1 expression, further decrease Aβ peptide formation, and inhibit amyloidogenic APP processing in AbetaPP/PS1 transgenic mouse brain [134,135]. P3 is a water-soluble copper chelator. P3 can specifically remove copper ions from the Cu-Aβ complex and prevent ROS production [136]. Trientine is a highly selective copper chelator. This drug can reduce copper overload in diabetic patients and inhibit ROS production [137]. Trientine has been shown to inhibit Aβ peptide deposition in the brains of APP/PS1 AD mice, resulting in reduced synaptic loss in the brain [138]. However, this drug has poor penetration into the central nervous system [138]. D-penicillamine is a common copper chelator that can inhibit ROS production in AD patients by reducing cellular copper concentration. Although this drug cannot cross the blood–brain barrier, researchers developed nanoparticles to deliver it to brain tissue [139]. In this study, Cui et al. conjugated D-penicillamine to nanoparticles via disulfide bonds and showed that the released drug, upon reduction, effectively resolubilized copper-induced Aβ(1-42) aggregates in vitro [139]. Tetrathiomolybdate (TTM) enhanced spatial memory ability in mice by increasing ADAM10 expression and reducing copper ion and Aβ peptide concentrations. However, the ability of this drug to penetrate the blood–brain barrier remains to be determined [140,141]. Polygonatum sibiricum polysaccharide (PSP) has been shown to directly bind to the cuproptosis-related protein DLAT in 3×Tg-AD mice. PSP treatment also modulated the expression of cuproptosis-associated proteins, alleviated mitochondrial damage, and activated the PI3K/AKT signaling pathway, thereby improving cognitive function [142]. However, the precise molecular mechanism linking PSP-mediated DLAT targeting to the activation of the downstream PI3K/AKT pathway remains unclear and requires further experimental validation.

## 5. Copper and Parkinson’s Disease

### 5.1. Copper Homeostasis and Parkinson’s Disease

PD is a common neurodegenerative disease characterized by resting tremor, muscle rigidity, and motor dysfunction [158,159]. A hallmark feature of PD is the degeneration and death of dopaminergic neurons in the substantia nigra pars compacta of the midbrain and the consequent reduction in dopamine content in the striatum [160]. PD has a high incidence and high disability rates, and the number of cases has been rising in recent years [161].

Altered copper concentrations have been observed in patients with PD, indicating a disruption of copper homeostasis (Table 2). Epidemiological studies suggest that increased environmental copper ion exposure raises the risk of developing PD [162,163]. However, in patients, this systemic dysregulation manifests as decreased copper ion concentration in the brain’s substantia nigra [112]. Ceruloplasmin deficiency particularly affects the function of proteins related to neuronal projection extension, synaptic signaling, and cellular mRNA processing, as well as protein expression in PD mice [164,165].

**Figure 4:**
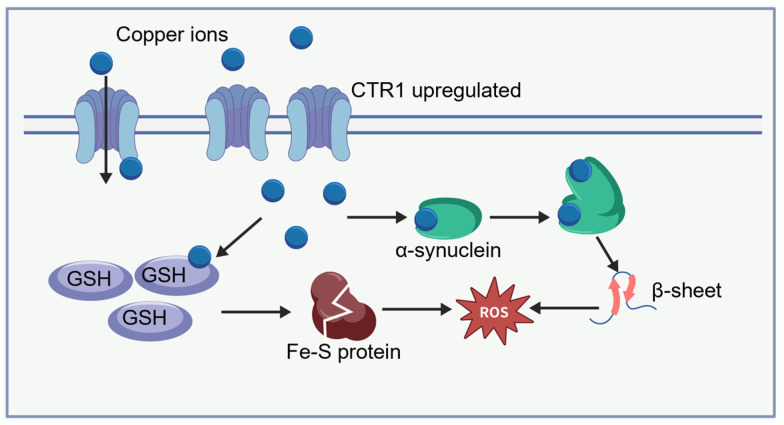
The potential mechanisms by which copper ions affect the progression of PD. The copper ions entering cells mediated by CTR1 can, in turn, act on CTR1 itself, resulting in an increase in CTR1 expression and intracellular copper content. On the one hand, Copper ions can bind α, causing it to aggregate and form a β-sheet structure. On the other hand, copper ions can bind to intracellular GSH, reducing its content and eventually causing the loss of Fe-S protein. Both of these effects lead to enhanced intracellular oxidative stress and promote the development of PD. This figure was created by the authors using BioGDP.com based on the literature cited in this review. In this figure, arrows indicate the direction of regulation or transport. Different colors are used to distinguish distinct cellular compartments, molecular pathways, and specific proteins as indicated in the figure.

A key mechanism linking copper homeostasis dysregulation to PD pathogenesis is the interaction between copper ions and α-synuclein, a core pathological protein in PD (Figure 4). α-synuclein is divided into three domains: the N-terminal domain, the hydrophobic non-amyloid-β component (NAC) domain, and the acidic C-terminal domain [166]. Studies indicate that α-synuclein has three copper ion binding sites: a high-affinity site at the N-terminal Met1-Asp2, and two lower-affinity C-terminal sites anchored to His50 and Asp121 residues, respectively [166]. Compared to the metal-free form, copper ion binding increases the neurotoxicity of α-synuclein in vitro [167]. Through multi-site interactions, copper alters the protein’s conformation and promotes its aggregation [168]. This copper-induced aggregation stimulates ROS production and triggers apoptosis [169]. Structural studies suggest that copper-induced α-synuclein aggregates form complexes involving two protein monomers, where the Met1-Asp2 domain and the His50 binding site of each monomer coordinate with a copper ion [170]. Binding of copper ions to both C-terminal and N-terminal sites reduces the polypeptide’s size and decreases structural flexibility in vitro [171]. Furthermore, binding at the N-terminus introduces new contact points between the N-terminal domain and the NAC domain, generating permanent new secondary structure elements, altering the protein’s structure, such as β-strands and hairpin structures [171]. The above structures lead to the formation of α-synuclein aggregates.

In a membrane simulation environment, copper ions (mainly Cu^+^) can coordinate with the N-terminal Met1 and Met5 residues of α-synuclein, forming a 1:2 complex bridging two α-helical peptide chains with a copper ion; this coordination mode stabilizes Cu^+^ and inhibits its catalytic activity for the oxidation of catechol substrates [172]. At the same time, copper ions can accelerate the prion-like propagation of α-synuclein fibers: it is mediated by negatively charged cell membrane heparan sulfate proteoglycans, promoting the internalization of α-synuclein fibers, enhancing intracellular aggregation, and facilitating the release of mature fibers to the extracellular space, triggering a new round of propagation [173]. The α-synuclein fibers formed in the presence of copper have stronger cytotoxicity, which can shorten the lifespan of the Caenorhabditis elegans PD model overexpressing human α-synuclein, and these toxic effects can be alleviated by copper chelators [169].

The binding affinity of α-synuclein for copper ions is further modulated by post-translational modifications [174]. Under physiological conditions, N-terminal acetylation of α-synuclein eliminates the Met1-Asp2 metal binding site, resulting in limited copper binding affinity [175]. The pathway of copper entry into cells exacerbates the detrimental effects of this PTM-induced reduction in copper binding affinity. After copper ions are transported into neuronal cells via CTR1, they upregulate the intracellular expression of CTR1 and further promote the aggregation and phosphorylation of α-synuclein in yeast and mammalian cell models [76]. Overexpression of CTR1 leads to increased cellular copper ion transport, causing intracellular copper accumulation. Studies show that knocking down CTR1 reduces phosphorylation of α-synuclein at serine 129, alleviates the loss of dopaminergic neurons, and mitigates α-synuclein-induced motor deficits in differentiated SH-SY5Y neuroblastoma cells [176].

Besides direct α-synuclein interaction, copper toxicity and impaired intracellular antioxidant systems mutually reinforce each other (Figure 4). In the brains of PD patients and animal models, copper toxicity is often inhibited by GSH, which chelates intracellular copper ions [12,177]. In PD tissues, as GSH levels drop significantly, copper ions accumulate intracellularly, disrupting the function of iron-sulfur cluster proteins and inducing oxidative stress, ultimately leading to cuproptosis [66]. This creates a vicious cycle: chronic copper exposure drives CTR1 overexpression, increased copper influx, and consequent GSH depletion in mice [178], which further exacerbates copper toxicity. Experimental evidence confirms that removing excess intracellular copper ions can reduce neuronal apoptosis and partially restore the expression of cuproptosis-related proteins in a mouse model [179]. Evidence also suggests that copper toxicity is associated with mitochondrial impairment in PD. In A53T α-syn transgenic mice, chronic low-dose copper exposure induced alterations in mitochondrial respiratory pathways, accompanied by impaired mitophagy and exacerbated α-syn accumulation. Consistently, low-dose Cu^2+^ treatment of A53T α-syn-overexpressing SH-SY5Y cells increased ROS production and reduced mitochondrial ATP generation, suggesting that even modest copper overload may contribute to mitochondrial dysfunction in PD [180].

Although no direct causal relationship between PD and cuproptosis has been established, at the molecular level, the expression of cuproptosis-related genes is altered in PD model cells, with DLAT and FDX1 being downregulated and CTR1, DBT, and ATP7A being upregulated (Table 3) [133].

### 5.2. Copper-Related Therapeutic Strategies in PD

Current research indicates that several copper chelators have potential therapeutic value for PD (Table 3). The copper chelator ATH434 restores patients’ motor and olfactory deficits by reducing intracellular copper ion concentration [144], which has not yet been verified by human experiments. D-penicillamine works by forming stable cyclic complexes with copper ions, thereby lowering copper ion levels and alleviating clinical manifestations in PD patients [181]. The chelator 1-methyl-1H-imidazole-2-carboxaldehyde isonicotinoyl hydrazone can competitively bind copper ions associated with α-synuclein, thereby reducing protein aggregation in H4 cells [145]. In the PD model, metformin restricts microglial cell activation by inhibiting the TLR4/MyD88/NF-κB pathway, thereby reducing copper ion levels and down-regulating cuproptosis-related proteins such as FDX1, SLC31A1, and heat shock protein 70 (HSP70) [146]. Ultimately, this leads to neuroprotective effects against cuproptosis.

CuIIATSM attenuates the misfolding and aggregation of wild-type SOD1 in the substantia nigra by replenishing regional copper deficiency, and this correction of SOD1 proteostasis is highly correlated with the protection of dopamine neurons, establishing brain copper supplementation as a potential strategy to slow neurodegeneration in Parkinson’s disease [148]. Whole-genome transcriptomic profiling in the MPTP-induced Parkinson’s disease mouse model revealed that CuIIATSM treatment restored the expression of 40 genes involved in dopamine synthesis, calcium signaling, and synaptic plasticity, providing the first mechanistic insight into its neuroprotective effects at the transcriptional level beyond its canonical copper-delivery function [147]. Also, a phase I trial in patients with early-stage PD established the recommended dose of CuIIATSM and demonstrated favorable tolerability (ClinicalTrials.gov database, NCT03204929). However, the efficacy outcomes from larger phase II/III clinical trials have not yet been officially published. Nevertheless, the currently available evidence provides an initial indication that modulation of copper homeostasis may represent a promising therapeutic strategy for PD.

## 6. Copper and Amyotrophic Lateral Sclerosis

### 6.1. Copper and ALS

ALS is a neurodegenerative disease characterized by insidious onset, rapid progression, and poor prognosis. Patients exhibit progressive degeneration of upper and lower motor neurons and atrophy of systemic striated muscle [182]. Most patients die within 5 years of diagnosis due to respiratory failure caused by respiratory muscle atrophy [1]. Statistics show that familial ALS (fALS) accounts for about 10–20% of total ALS cases, with the remainder being sporadic ALS (sALS) patients [1,183].

Multiple studies indicate that copper homeostasis is closely related to disease progression in ALS patients (Table 1). Research shows that the copper concentration in the cerebrospinal fluid of ALS patients is higher than in healthy individuals [115]. These results support the hypothesis of copper ion accumulation and its potential involvement in ALS pathogenesis [184]. However, another report indicated that copper levels in the cerebrospinal fluid of ALS patients, particularly those with spinal onset, were lower than in normal individuals and patients with bulbar onset [116]. These inconsistent findings regarding cerebrospinal fluid copper levels may stem from differences in patient cohorts, disease stages, or methodologies. Nonetheless, they consistently point to severe dysregulation of copper homeostasis in ALS. In a transgenic mouse model of ALS expressing the human pathogenic mutant hSOD1^G93A^, reduced copper content in the spinal cord and skeletal muscle can be detected even before ALS onset, and this trend persists with disease development [113,114].

**Figure 5:**
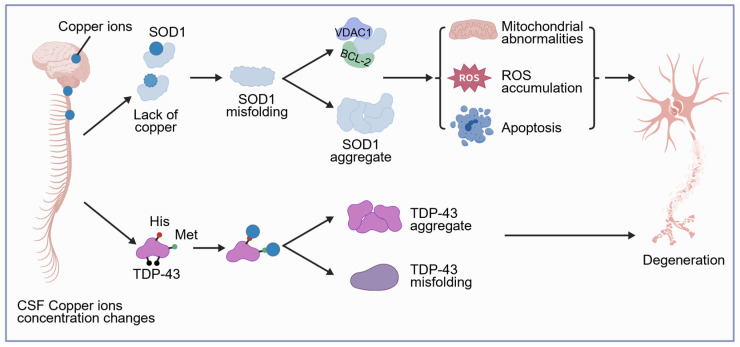
Dysregulation of copper homeostasis mediates motor neuron injury through two pathways, SOD1 and TDP-43. Mutations in SOD1 protein result in improper binding of copper ions, resulting in SOD1 misfolding and the formation of insoluble aggregates. These misfolded SOD1 proteins can bind to mitochondrial outer membrane receptors such as BCL-2 and VDAC1, ultimately inducing mitochondrial dysfunction and activating oxidative stress or apoptotic pathways, which in turn cause neuronal death. In addition, copper ions also directly interact with TDP-43: TDP-43 binds Cu^2+^ through histidine and methionine residues and promotes sulfhydryl oxidation of Cys173 and Cys175 to form disulfide bonds in the RRM1 domain, thereby inducing TDP-43 misfolding and enhancing its aggregation propensity, triggering neurotoxicity and ultimately leading to motor neuron injury. This figure was created by the authors using BioGDP.com based on the literature cited in this review. In this figure, arrows indicate the direction of regulation or transport. Different colors are used to distinguish distinct cellular compartments, molecular pathways, and specific proteins as indicated in the figure.

Degeneration of upper and lower motor neurons is a significant factor in ALS development. Current research indicates that mutations in SOD1 play an important role in motor neuron degeneration [185] and represent one of the primary mechanisms in fALS pathogenesis [185]. Studies show that misfolded SOD1 protein caused by G93A or H46R mutations can bind to some mitochondrial outer membrane receptors, such as BCL-2 and VDAC1, ultimately leading to mitochondrial dysfunction, oxidative stress, or activation of apoptotic pathways, resulting in neuronal cell death in a mouse model (Figure 5) [186,187,188,189]. The correct binding of SOD1 with copper ions is crucial for its full physiological function (Table 4). Research indicates that disruption of intracellular copper homeostasis exacerbates the misfolding (e.g., hSOD1^G93A^) and formation of insoluble aggregates of SOD1 in human spinal cord tissue (Figure 5) [190]. Simultaneously, the SOD1 A4V mutation reduces enzyme activity and its copper affinity [191]. Interestingly, in multiple SOD1 protein mutants (G37R, G93A, H46R, H48Q, A4V, H80R, D125H mutants), reduced copper ion binding to the protein was detected, and this was independent of the strength of copper binding ability of the mutant protein in cells and mouse models [192,193]. Furthermore, the degree of copper deficiency in SOD1 aggregates correlates with the disease severity of patients [194].

Additionally, the copper transport chaperone CCS, which delivers copper to SOD1, also plays an important role in ALS disease development. In the hSOD1^G93A^ transgenic ALS mice model, overexpression of CCS reduced survival [195], which can be rescued by copper delivery complexes [198]. Interestingly, the situation seems opposite in ALS patients: higher CCS expression is positively correlated with longer survival [199]. The reason for this discrepancy is not yet clear and may be related to species differences between mice and humans, and research on the relationship between cuproptosis and ALS is currently lacking.

Additionally, copper has been shown to interact with TDP-43, a central mediator of ALS pathogenesis. The misfolding, aggregation, and aberrant accumulation of TDP-43 in neurons are recognized as hallmark pathological features of the disease [200]. Previous studies have shown that TDP-43 is capable of binding Cu^2+^ through histidine and methionine residues, promoting thiol oxidation of Cys173 and Cys175 to form disulfide bonds within the RRM1 domain [196]. These copper-mediated modifications potentially alter the conformation and functional properties of TDP-43, thereby influencing its misfolding and aggregation propensity. Such findings support the notion that copper dyshomeostasis contributes to TDP-43-mediated neurotoxicity (Figure 5) [196]. However, other studies have demonstrated that treatment with bis (thiosemicarbazonato)-copper complexes can reduce TDP-43 accumulation in SH-SY5Y cells [197]. These observations suggest that the biological effects of copper are not unidirectionally toxic. Rather, the pathological or protective role of copper likely depends on its chemical form or delivery mechanism (Figure 5).

### 6.2. Copper-Related Therapeutic Strategies in ALS

The disruption of copper homeostasis in ALS has prompted the exploration of therapeutic strategies using copper chelators or copper ionophores (Table 3). Several copper chelators have been evaluated for their effects. Copper delivery agents (CuIIATSM) and copper chelators (such as TTM and D-penicillamine) have shown therapeutic potential in ALS [150,151]. D-penicillamine is a copper chelator that inhibits the peroxidase activity of mutant SOD1, thereby delaying disease onset and extending survival in ALS mouse models [155]. Trientine, also a copper chelator, attenuates copper-mediated oxidative toxicity derived from mutant SOD1 and has been shown to produce a modest but significant increase in survival in transgenic ALS mice [156]. CuIIATSM treatment improved motor function and survival in hSOD1^G93A^ and hSOD1^G93R^ transgenic mice [150,153,201]. In the hSOD1^G93R^ transgenic mice, CuIIATSM increased copper delivery to the metal-deficient apo-form of SOD1 and promoted its conversion to the more stable fully metallated holo-SOD1 form [150]. Although CuIIATSM treatment led to increased SOD1 protein concentration in hSOD1^G37R^ transgenic mice, it still had a therapeutic effect, extending mouse survival [150]. Oral administration of CuIIATSM delayed disease onset, improved locomotor function, and extended survival in hSOD1^G93A^ ALS mice, associated with increased levels of mature mutant SOD1 protein, copper content, and SOD1 enzymatic activity in the spinal cord in a CNS-selective manner [153]. However, clinical studies of CuIIATSM in ALS have yielded less encouraging results. A phase I clinical trial investigating orally administered CuIIATSM in patients with ALS has been conducted (ClinicalTrials.gov database, NCT02870634). However, subsequent postmortem analyses revealed that CuIIATSM treatment did not significantly alter motor neuron pathology in either the motor cortex or spinal cord [152]. These findings suggest that the therapeutic benefits of CuIIATSM in clinical settings may be more limited than those observed in preclinical animal models.

TTM can prolong the survival time of hSOD1^G93A^ transgenic mice, reduce motor neuron loss, and alleviate muscle atrophy symptoms [150]. Mechanistically, TTM can inhibit the aggregation activity of mutant SOD1, reducing its aggregation [149]. Furthermore, the copper chelators D-penicillamine and trientine have also been shown to extend the survival time of ALS mouse models [154,155]. Additionally, a bioinformatics analysis suggested that panobinostat, olaparib, and apilimod may have potential therapeutic value for ALS; however, the effects and mechanisms of these drugs remain to be determined [157].

## 7. Conclusions and Perspectives

Copper dysregulation has emerged as a consistent feature across major neurodegenerative diseases, including AD, PD, and ALS [28]. The present review integrates current evidence to highlight that, although copper imbalance is closely associated with mitochondrial dysfunction, protein misfolding, and neuronal vulnerability, the precise mechanisms linking copper dysregulation to neuronal loss remain incompletely defined. In this context, cuproptosis provides a mechanistically defined framework for copper-dependent cytotoxicity; however, its pathological relevance in neurodegeneration requires careful evaluation.

A critical issue in the current literature is the distinction between cuproptosis and general copper-associated cellular stress. Mechanistically, cuproptosis is defined by copper-dependent aggregation of lipoylated mitochondrial proteins and destabilization of Fe-S cluster proteins. However, most studies in neurodegenerative disease models have primarily reported alterations in copper distribution, redox imbalance, or changes in the expression of cuproptosis-related genes [132]. Although informative, these observations do not constitute direct evidence of cuproptosis. Therefore, distinguishing between dysregulation of the cuproptosis machinery and bona fide activation of cuproptosis is essential for accurate mechanistic interpretation.

Another key consideration is the metabolic dependency of cuproptosis. Previous studies indicate that susceptibility to cuproptosis is tightly linked to mitochondrial oxidative phosphorylation and TCA cycle activity [66]. Neurons are highly energy-demanding cells with substantial reliance on mitochondrial metabolism; however, metabolic heterogeneity exists across neuronal subtypes and disease states. It remains unclear whether vulnerable neuronal populations in AD, PD, and ALS exhibit the specific metabolic configurations required to support cuproptosis. Addressing this question will be critical for determining whether cuproptosis represents a primary driver of neurodegeneration or a context-dependent secondary process.

Another important unresolved question is whether cuproptosis is a reversible or irreversible process, as this has important implications for therapeutic intervention. It is conceivable that early events, such as intracellular copper accumulation and disruption of copper homeostasis, may be reversible if copper levels are restored before extensive cellular damage occurs [153]. However, whether later events-including aggregation of lipoylated proteins, loss of iron–sulfur cluster proteins, and mitochondrial dysfunction-represent an irreversible commitment to cell death remains to be determined. Addressing this question may be particularly relevant for neurodegenerative diseases, where pathological changes develop gradually over many years. A better understanding of the reversibility of cuproptosis could help define the optimal timing for therapeutic intervention and guide the development of more effective copper-targeting strategies. Future studies are therefore needed to identify biomarkers that distinguish potentially reversible stages from irreversible cellular damage.

The disease-specific evidence further underscores this uncertainty. In AD, copper-amyloid interactions and extracellular copper accumulation are well documented but are largely spatially and mechanistically distinct from mitochondrial cuproptosis [96]. In PD, discrepancies between systemic copper exposure and reduced copper levels in the substantia nigra suggest complex, compartment-specific dysregulation rather than a uniform increase in intracellular copper toxicity [112,163]. In ALS, mutations in copper-binding proteins such as SOD1 clearly implicate copper handling in disease pathogenesis; however, direct links to lipoylated protein aggregation or Fe-S cluster instability have not been established [192]. Collectively, these observations support a model in which copper dysregulation contributes to neurodegeneration through multiple mechanisms, with cuproptosis representing a plausible but yet unverified pathway.

From a therapeutic perspective, strategies targeting copper metabolism, including chelators and copper-delivering agents, have shown potential in preclinical models [142]. However, the lack of mechanistic clarity poses a challenge for rational intervention. Without definitive evidence that cuproptosis is operational in disease-relevant neuronal populations, it remains uncertain whether modulating this pathway will yield therapeutic benefit. Future studies should therefore prioritize the identification of core hallmarks of cuproptosis in vivo, including the aggregation of lipoylated mitochondrial proteins, the loss of Fe-S cluster proteins, and the dependency on mitochondrial metabolic state.

Despite the encouraging therapeutic potential of copper-targeting strategies in neurodegenerative diseases, their clinical application also presents important challenges and potential risks. Copper is an essential trace element that serves as a cofactor for numerous enzymes involved in mitochondrial respiration, antioxidant defense, neurotransmitter biosynthesis, and other fundamental cellular processes [28]. Therefore, excessive depletion of copper may impair normal cellular physiology and compromise neuronal function. Conversely, therapeutic approaches aimed at increasing intracellular copper availability, such as CuIIATSM, may under certain conditions promote excessive copper accumulation, thereby increasing the risk of copper toxicity or aberrant activation of cuproptosis. These considerations highlight that the therapeutic objective should not simply be to remove or supplement copper, but rather to restore and maintain copper homeostasis within its physiological range. Future studies should focus on developing precision copper-modulating strategies that achieve spatiotemporal regulation of copper metabolism while minimizing off-target effects and preserving normal copper-dependent biological functions. In particular, the safety and potential neurotoxicity of copper-targeting agents require careful evaluation to ensure their successful translation into therapies for neurodegenerative diseases.

Several important challenges remain before the pathological relevance of cuproptosis in neurodegenerative diseases can be fully established. First, current evidence is derived predominantly from immortalized cell lines and animal models, whereas direct validation in human neuronal tissues remains limited. Future studies employing human brain samples and patient-derived neuronal models will therefore be essential to determine whether cuproptosis occurs under pathological conditions. Second, many experimental models rely on supraphysiological copper exposure or synthetic copper ionophores, which may not accurately reflect the gradual and heterogeneous copper dyshomeostasis observed in neurodegenerative diseases. Developing physiologically relevant models that better recapitulate neuronal metabolism and disease progression will be critical. Third, the currently recognized molecular signature of cuproptosis is largely based on lipoylated protein aggregation and iron–sulfur cluster protein loss. Additional molecular markers are needed to improve the identification and characterization of cuproptosis in complex tissues. Finally, integrative approaches combining metabolomics, proteomics, spatial imaging, and single-cell technologies may provide a more comprehensive understanding of copper redistribution and cuproptosis during disease progression.

In summary, while copper dysregulation is a well-established feature of neurodegenerative diseases, the contribution of cuproptosis remains to be conclusively determined. Current evidence supports a model in which copper imbalance reshapes mitochondrial function and cellular metabolism, thereby modulating neuronal vulnerability. Whether this process culminates in cuproptosis is an open question that warrants rigorous experimental investigation. Clarifying this distinction will be essential for advancing our understanding of neurodegenerative disease mechanisms and for developing targeted therapeutic strategies.

## Figures and Tables

**Table 2:** Copper homeostasis- and cuproptosis-related gene expression changes in AD and PD.

Disease	Genes	Tissue	Expression	Reference
AD	CTR1/ATP7B/LIPT1 CDKN2A/MTF1	Cerebral cortex	Upregulated ↑	[132]
	FDX1/DLAT/GLS/PDHA1/DLD PDHB/LIAS	Cerebral cortex	Downregulated ↓	[132]
	CTR1, DBT, and ATP7A	Substantia Nigra	Upregulated ↑	[133]
PD	DLAT and FDX1	Substantia Nigra	Downregulated ↓	[133]

**Table 3:** Copper-related therapeutic strategies in AD, PD, and ALS.

Disease	Drug	Drug Properties	Blood–Brain Barrier Permeability	Experimental Evidence	Clinical Status	Mechanism	Reference
AD	Clioquinol	Copper ionophore and chelator	+	Animal/Open-label exploratory study	Preclinical	Inhibits Aβ aggregation and BACE1 expression	[134,135,143]
	P3	Copper chelator	Unknown	In Vitro	Preclinical	Removes copper from Cu-Aβ complex, prevents ROS production	[136]
	Trientine	Copper chelator	−	Animal/SH-SY5Y Cells	Precilinical	Inhibits ROS and Aβ deposition	[137,138]
	D-penicillamine	Copper chelator	−	In Vitro	Preclinical	Increases ADAM10 expression, inhibits Aβ production and aggregation	[139]
	TTM	Copper chelator	Unknown	Animal/N2a- sw Cells	Preclinical	Increases ADAM10 expression, reduces copper and Aβ levels	[140,141]
	Polygonatum Sibiricum polysaccharide	Copper chelator	+	Animal/HT22 cells	Preclinical	Targets DLAT, activates the PI3K/AKT pathway, and modulates cuproptosis-related proteins to alleviate mitochondrial damage	[142]
	ATH434	Iron chelator	+	Animal	Preclinical	Reduces intracellular copper concentration	[144]
PD	1-methyl-1H-imidazole-2-carboxaldehyde isonicotinoyl hydrazone	Copper chelator	Unknown	Human neuroglioma (H4) cells	Preclinical	Competitively binds α-synuclein-associated copper ions, inhibiting protein aggregation	[145]
	Metformin	Copper-sequestering agent	+	Animal/BV2 cell	Phase III	Inhibits the TLR4/MyD88/NF-κB pathway to downregulate copper ion levels and cuproptosis-related proteins	[146]
	CuIIATSM	Copper ionophore	+	Animal/Clinical Trial (NCT03204929)	Phase I	Restores wild-type SOD1 function and corrects gene expression networks involved in dopamine synthesis, calcium signaling, and synaptic plasticity.	[147,148]
	TTM	Copper chelator	Unknown	Animal	Preclinical	Inhibits mutant aggregation	[149]
ALS	CuIIATSM	Copper ionophore	+	Animal/Clinical trial (NCT04082832)	Phase III	Promoting copper metallation of mutant SOD1; CNS SOD1 restoration, prolongs lifespan	[150,151,152,153]
	D-penicillamine	Copper chelator	Unknown	Animal	Preclinical	Inhibits peroxidase activity of mutant SOD1	[154,155]
	Trientine	Copper chelator	−	Animal	Preclinical	Attenuates copper-mediated oxidative toxicity derived from mutant SOD1	[154,155,156]
	Panobinostat/Olaparib/Apilimod	Anticancer agent/anticancer agent/immunomodulator	Controversial/ −/Unknown	Bioinformatics analysis	Preclinical/Preclinical/Phase II	Undetermined	[157]

**Table 4:** The relationship between different ALS pathogenic proteins and copper ions.

SOD1 Mutant	Mechanism	References
SOD1 A4V	Lowers enzyme activity and copper affinity; reduced Cu binding.	[192,193]
SOD1 H46R	Misfolding leads to mitochondrial damage via BCL-2/VDAC1; reduced Cu binding.	[192,193]
SOD1 G37R/H48Q H80R/D125H	Reduced copper binding, independent of inherent copper affinity.	[192,193]
SOD1 G93A	Copper dyshomeostasis promotes aggregation; reduced copper binding.	[195]
TDP-43	Cu promotes the oxidation of thiol groups to form disulfide bonds	[196]
TDP-43	Treatment with bis(thiosemicarbazonato)-copper complexes reduces TDP-43 accumulation	[197]

## Data Availability

No new data were created or analyzed in this study. Data sharing is not applicable to this article.

## Abbreviations

Aβ	amyloid-β
AD	Alzheimer’s disease
ADAM10	a disintegrin and metalloproteinase domain 10
ALA	alpha-lipoic acid
ALS	amyotrophic lateral sclerosis
APP	amyloid precursor protein
ATM1	ABC transporter of the mitochondrion 1
ATOX1	antioxidant 1 copper chaperone
ATP7A	ATPase copper-transporting α polypeptide
ATP7B	ATPase copper-transporting β polypeptide
BACE1	β-site APP cleaving enzyme 1
BDNF	brain-derived neurotrophic factor
COA6	cytochrome c oxidase assembly factor 6
COX1	cytochrome c oxidase subunit 1, also known as MT-CO1
COX2	cytochrome c oxidase subunit 2, also known as MT-CO2
COX11	cytochrome c oxidase assembly protein 11
COX16	cytochrome c oxidase assembly protein 16
COX17	cytochrome c oxidase copper chaperone 17
CCS	copper chaperone for SOD1
CRGs	cuproptosis-related genes
CTR1	copper transporter 1, also known as SLC31A1
CTR2	copper transporter 2, also known as SLC31A2
CuL	low-molecular-weight anionic ligand complex
DBH	dopamine-β-hydroxylase
DBT	dihydrolipoamide branched chain transacylase
DCYTB	duodenal cytochrome B
DLAT	dihydrolipoamide S-acetyltransferase
DLST	dihydrolipoamide S-succinyltransferase
fALS	familial ALS
FDX1	ferredoxin 1
Fe-S	iron-sulfide
GABAA	gamma-aminobutyric acid type A
GCSH	glycine cleavage system H protein
GSH	glutathione
HO-1	heme oxygenase-1
HSP70	heat shock protein 70
IL-1β	interleukin-1β
IL-6	interleukin-6
iNOS	inducible nitric oxide synthase
LA	lipoic acid
LIAS	lipoic acid synthetase
LRP1	low-density lipoprotein receptor-related protein 1
MPTP	1-methyl-4-phenyl-1,2,3,6-tetrahydropyridine
NAC	non-amyloid-β component
NMDA	N-methyl-D-aspartate
NMDAR	N-methyl-D-aspartate receptor
NQO1	NAD(P)H quinone dehydrogenase 1
Nrf2	nuclear factor erythroid 2-related factor 2
PAM	peptidylglycine α-amidating monooxygenase
PD	Parkinson’s disease
PSP	Polygonatum Sibiricum polysaccharide
ROS	reactive oxygen species
sALS	sporadic ALS
SCO1	synthesis of cytochrome c oxidase 1
SCO2	synthesis of cytochrome c oxidase 2
SOD1	copper-zinc superoxide dismutase 1
STEAP	six-transmembrane epithelial82antigen of the prostate
TCA	tricarboxylic acid
TGN	trans-Golgi network
TTM	tetrathiomolybdate
TNF-α	tumor necrosis factor-α
VDAC1	voltage-dependent anion channel 1°
